# Efficacy of Yigu® versus Aclasta® in Chinese postmenopausal women with osteoporosis: a multicenter prospective study

**DOI:** 10.1007/s11657-021-01052-y

**Published:** 2022-01-12

**Authors:** Mei Li, Qun Cheng, Ya-nan Huo, Ai-jun Chao, Liang He, Qing-yun Xue, Jin Xu, Shi-gui Yan, Hui Jin, Zhen-lin Zhang, Jian-hua Lin, Xiao-lan Jin, You-jia Xu, Feng Liu, Wei-bo Xia

**Affiliations:** 1 Department of Endocrinology, Peking Union Medical College Hospital, Beijing, China; 2 Department of Osteoporosis and Bone Disease, Huadong Hospital Affiliated To Fudan University, Shanghai, China; 3 Department of Endocrinology, Jiangxi People’s Hospital, Jiangxi, China; 4 Department of Cadre Ward, Tianjin Hospital, Tianjin, China; 5 Orthopedics Department, Beijing Jishuitan Hospital, Beijing, China; 6 Department of Orthopedics, Beijing Hospital, Beijing, China; 7 Department of Endocrinology, Shandong Provincial Hospital, Jinan, China; 8 Department of Orthopaedic Surgery, The Second Affiliated Hospital, School of Medicine, Zhejiang University, Zhejiang, China; 9 Department of Endocrinology, The Affiliated Zhongda Hospital of Southeast University, Nanjing, China; 10 Department of Osteoporosis and Bone Diseases, Shanghai Jiao Tong University Affiliated Sixth People’s Hospital, Shanghai, China; 11 Department of Orthopedic Surgery, The First Affiliated Hospital of Fujian Medical University, Fuzhou, China; 12 Department of Endocrinology, General Hospital of People’s Liberation Army Chengdu Military Region, Chengdu, China; 13 Orthopaedic Department, The 2nd Affiliated Hospital of Suzhou University, Suzhou, China; 14 Department of Geriatric, The First Municipal People’s Hospital of Guangzhou, Guangzhou, China

**Keywords:** Zoledronic acid, Postmenopausal osteoporosis, BMD, β-CTX, P1NP

## Abstract

***Summary*:**

Zoledronic acid (ZOL) is a therapy inhibiting bone resorption. In this study, generic ZOL (Yigu®) showed its clinical efficacy consistency with original ZOL (Aclasta®) in Chinese postmenopausal women with osteoporosis. This study provides a practical basis for the application of Yigu® in Chinese population.

**Introduction:**

Yigu® has been approved its bioequivalence to Aclasta®. However, the clinical efficacy and safety of Yigu® have not been evaluated yet. Here, we compared the effectiveness and safety between Yigu® and Aclasta® in Chinese postmenopausal women with osteoporosis and assessed the efficacy of intravenous infusion of ZOL.

**Methods:**

This was a randomized open-label, active-controlled study in postmenopausal women with osteoporosis of 14 clinical centers in China. Postmenopausal women with osteoporosis were recruited and randomized to receive a single infusion of 5 mg Yigu® or Aclasta®. The primary endpoint was the percentage change in bone mineral density (BMD) at lumbar spine after 12 months of treatment and was assessed for equivalence. The secondary endpoint was the percentage change in BMD at proximal femur after 12 months. Additional secondary endpoints were percentage changes in BMD at the above sites after 6 months of treatment and changes in bone turnover biomarkers during ZOL treatment. Safety was also evaluated and compared between two groups.

**Results:**

A total of 458 postmenopausal women with osteoporosis were enrolled (*n* = 227, Yigu®; *n* = 231, Aclasta®). The mean percentage change in the BMD had no statistical difference at the lumbar spine (5.32% vs 5.18%), total hip (2.72% vs 2.83%), and femoral neck (2.37% vs 2.81%) between Yigu® and Aclasta® groups after 12 months of treatment. The mean difference of BMD change at the lumbar spine after 12 months between two groups was 0.15% (95% CI: − 0.71 to 1.00, equivalence margin: − 1.5%, 1.5%), demonstrating the treatments were equivalent. Meanwhile, the decreases in the P1NP and β-CTX showed no difference between two groups after 14 days and 6 and 12 months of treatment. As regards the whole sample, BMD significantly increased after 12 months of treatment. Also, serum C-terminal telopeptide of type 1 collagen (β-CTX) and procollagen 1 N-terminal peptide (P1NP) significantly decreased at each visit period. The overall adverse events were comparable and quite well between two groups.

**Conclusion:**

Intravenous infusion of zoledronic acid achieved the potent anti-resorptive effects which led to significant increase in BMD of Chinese postmenopausal women with osteoporosis. Yigu® was equivalent to Aclasta® with respect to efficacy and safety.

## Introduction

Osteoporosis is a chronic, progressive disease characterized by low bone mass and deterioration of bone micro-architecture, with a consequent increase in bone fragility and fracture susceptibility [1, 2]. According to the latest nationwide epidemiological survey of osteoporosis in 2018, 19.2% of the population over 50 years old and 32.0% of the population over 65 years old suffer from osteoporosis in China [3]. However, with the expansion of number of patients with osteoporosis, the costs of osteoporosis treatment are also increasing annually, which puts great medication burden on healthcare system. According to the report of National Health Commission of China in 2018, the expense of drugs has accounted for 30–40% of the national total health expenditures [4], which was higher than that of other countries [5]. Additionally, for patients in rural areas, although their medical insurance has been covered, they still need to bear a higher self-payment of drugs, especially original branded drugs, so they would rather not receive treatment.

Generic drugs are therapeutically equivalent to their high-priced branded counterpart, but much cheaper. Therefore, generic drugs play an undeniably important role in lowering national healthcare burden. Reportedly, generic drugs are estimated to save $2 trillion drug costs by 2028 [6]. Consequently, generic drugs are considered indispensable substitutes for high-priced original drugs.

Generic zoledronic acid (ZOL, Yigu®), a long-acting intravenous bisphosphonate, has been approved its bioequivalence to the original ZOL (Aclasta®). However, the clinical efficacy and safety of Yigu® have not been evaluated yet. Thus, it is of great urgency to develop its clinical efficacy consistency with Aclasta®. Meanwhile, Yigu® has been available in Chinese patients with osteoporosis already; however, some patients still concerned about the efficacy and safety of Yigu® due to the deep-rooted concept that lower price of drugs are associated with worse effects. Therefore, the objective of this study was to compare the efficacy and safety of Yigu® and Aclasta® in postmenopausal women with osteoporosis in China and to assess the efficacy and safety of annual infusion of ZOL.

## Material and methods

### Study design and treatment

The Postmenopausal Osteoporotic Women Efficacy and Safety Research (POWER) was a 12-month prospective, multicenter, randomized, open-label, active-controlled trial conducted at 14 clinical centers in China between June 2017 and March 2020. The study protocol was reviewed and approved by ethics committee of clinical pharmacology center of Peking Union Medical College Hospital (PUMCH) and all other participating units. This study was registered at ClinicalTrials.gov via Protocol Registration and Results System (PRS) on 21 May 2017. All patients were informed of detailed information about the study and signed written informed consents prior to enrollment.

All subjects were randomly assigned to receive a single infusion of either Yigu® (5 mg/100 mL, Chia Tai Tianqing Pharmaceutical Group Co., Ltd, China) or Aclasta® (5 mg/100 mL, Novartis Pharma Stein AG, Switzerland) in a 1:1 ratio by centralized random allocation system. In addition, all subjects were supplemented with 600 mg elemental calcium (Caltrate®, Wyeth Pharmaceutical Co., Ltd.) and 800 IU vitamin D (Xingsha®, Sinopharm Xingsha Pharmaceuticals (Xiamen, Co., Ltd.)) daily. Antipyretic analgesics, such as acetaminophen or ibuprofen, were allowed to alleviate the acute phase response (APRs) related to ZOL infusion [7–9].

### Subjects

Postmenopausal women aged between 45 and 80 years were eligible for inclusion if they had a BMD T-score of − 2.5 or lower at the lumbar spine, total hip, or femoral neck or a BMD T-score of − 1.0 or less at the lumbar spine, total hip, or femoral neck, with the history of fragility fracture of vertebra, hip, proximal humerus, or distal radius.

Exclusion criteria were as follows: abnormal hepatic function and renal function with alanine transaminase (ALT) or aspartate transaminase (AST) more than 2 folds of the upper limit of normal and plasma creatinine and urea nitrogen more than 1.5 folds of the upper limit of normal or calculated creatinine clearance less than 60 mL/min; serum calcium levels higher than 2.75 mmol/L (11.0 mg/dL) or less than 2.00 mmol/L (8.0 mg/dL); severe hematological or mental diseases; cancer or other serious progressive diseases; treatment history of bisphosphonates within recent 12 months before entering the study; treatment history of parathyroid hormone 1–34 or 1–84, estrogen, selective estrogen receptor modulators, and strontium more than 2 weeks within recent 6 months; and treatment history of glucocorticoid more than 3 months within recent 6 months. Additional exclusion criteria included allergy to study drugs and their metabolites, participating in other clinical studies within recent 3 months, or unfit for this trial based on investigator judgment.

### Effectiveness evaluation

The lumbar spine, total hip, and femoral neck BMD were measured using dual-energy X-ray absorptiometry (DXA) at baseline and 6 and 12 months of ZOL infusion with GE Lunar iDXA (GE Healthcare, Madison, WI) or Hologic Discovery (Hologic, Bedford, MA) in each clinical center. The quality control and BMD assessments were performed uniformly by radiologists of PUMCH.

Serum samples were collected after an overnight fast. Serum levels of bone turnover biomarkers including C-terminal telopeptide of type 1collagen (β-CTX) and procollagen 1 N-terminal peptide (P1NP) were measured at baseline, 14 days, and 6 and 12 months after ZOL infusion. All serum samples were transported to the central laboratory of PUMCH for unified storage and tested with cobas e 801 automatic chemiluminescence immunoassay analyzer (Roche Diagnostics, Risch-Rotkreuz, Switzerland). Minimum detectable value for β-CTX and P1NP was 0.05 ng/mL and 10 ng/mL, respectively, with an intermediate precision CV ≤ 20%.

The primary endpoint of this study was the percentage change in BMD at lumbar spine from baseline to 12 months of treatment. The secondary endpoint was the percentage changes in BMD at total hip and femoral neck from baseline to 12 months of treatment. Other secondary endpoints were the percentage changes in BMD at the above sites from baseline to 6 months of treatment and changes in β-CTX and P1NP levels from baseline to 14 days and 6 and 12 months of ZOL treatment.

### Safety evaluation

Safety of ZOL was assessed by clinical records and laboratory tests. Adverse events (AEs) were recorded through either follow-up visits or self-report. All AEs were coded using the Medical Dictionary for Regulatory Activities (MedDRA). Investigators interviewed subjects every 3 months to access information about AEs and concomitant medications. The body temperatures within 3 days after ZOL infusion and daily concomitant medications of subjects were recorded on report cards by the patients.

### Statistical analysis

In order to compare the efficacy and safety of Yigu® and Aclasta®, we utilized the equivalent hypothesis to demonstrate equivalence between the Yigu® and Aclasta® for the primary efficacy endpoint. It was assumed that an expected change of BMD at lumbar spine was 4.0% at month 12 for each group; a sample size of 194 patients per group would provide 80% statistical power to test the equivalence of two groups, with a two-sided significance level of 0.05 and equivalence margin of − 1.5%, 1.5%. Considering the 20% dropout rate, 233 subjects were designed to enroll in each group, for a total of 466 subjects.

All efficacy analyses were conducted on the full analysis set (FAS), which included all randomly assigned patients who received one infusion of ZOL. Randomization was computer-generated and was stratified by BMD measurement site and history of fragility fractures. The measurement data of each visit were statistically described as mean ± SD. Changes in BMD and bone turnover biomarkers between baseline and each visit period were analyzed using paired sample *t*-test. The changes in BMD and bone turnover markers before and after treatment between two groups were compared using analysis of covariance (ANCOVA) adjusted for baseline covariates (i.e., history of fragility fractures, trial center). The count data of each visit were statistically described by frequency (composition ratio), and changes before and after treatment were tested by *χ*^2^ test or non-parametric test.

The percentage change of lumbar spine BMD from baseline to 12-month treatment was tested for equivalence, and the equivalent threshold was 1.5%. The analysis of variance was used to evaluate the efficacy index. Since this study was a multicenter clinical study, the central effects were considered.

The safety analysis set (SAS) included all subjects who received one dose of study treatment. The Fisher’s exact test was used to compare the incidence of AEs between the Yigu® and Aclasta® groups.

The statistical analyses were carried out using SAS9.4 statistical software. All statistical tests used two-sided tests, and *P* < 0.05 was considered statistically significant.

## Results

### Demographic and baseline characteristics

**Fig. 1:**
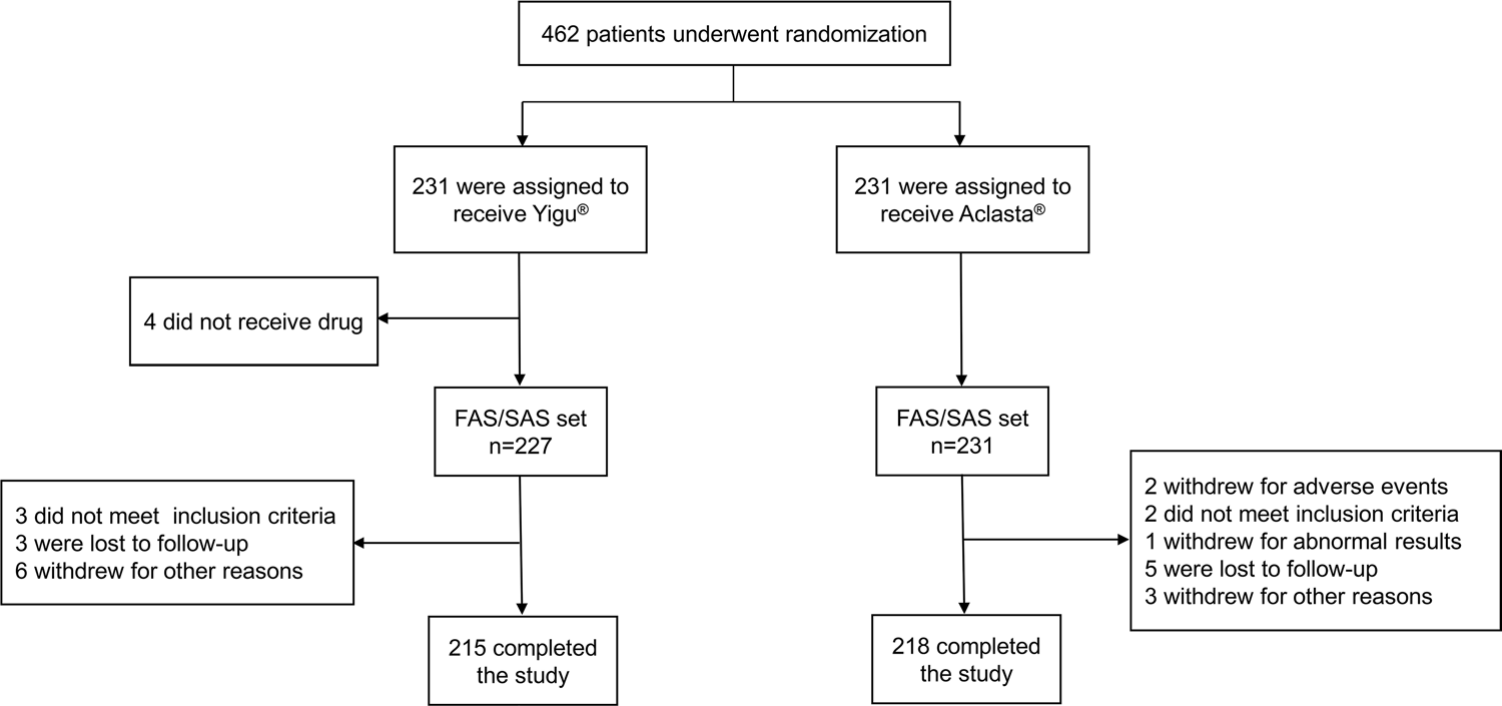
Flow chart of subject distribution. FAS full analysis set, SAS safety analysis set

A total of 462 subjects were included in the study (Fig. 1), and 458 subjects received one infusion of ZOL (Yigu®: *n* = 227, Aclasta®: *n* = 231). Two subjects withdrew early due to the AEs. Five subjects did not meet the inclusion criteria. One subject had hypocalcemia after the infusion. Eight subjects were lost to follow-up, and 9 subjects withdrew from the study for other reasons, including receiving other anti-osteoporotic drugs, voluntary withdrawal, and refusal to complete follow-up on time. Four hundred thirty-three subjects (93.72%) (Yigu®: *n* = 215; Aclasta®: *n* = 218) completed the 12 months of follow-up. The demographic and baseline characteristics were analyzed including 458 subjects based on the FAS (Table 1). No differences were observed between two groups in the baseline characteristics (Table 1).

**Table 1:** Demographic characteristics of subjects at baseline

Variable	ZOL (*n* = 458)	Yigu® (*n* = 227)	Aclasta® (*n* = 231)	*P* value between groups
Age (years)	64.32 ± 6.10	64.12 ± 6.10	64.52 ± 6.10	0.483
Height (m)	1.56 ± 0.06	1.56 ± 0.06	1.56 ± 0.06	0.245
Weight (kg)	56.80 ± 8.26	56.83 ± 8.44	56.76 ± 8.09	0.932
BMI (kg/m^2^)	23.40 ± 3.28	23.50 ± 3.27	23.30 ± 3.30	0.521
Menopausal period (years)	15.98 ± 11.15	15.28 ± 7.01	16.68 ± 14.05	0.180
Concomitant diseases, *n* (%)	289 (63.10)	150 (66.08)	139 (60.17)	0.209
Hypertension	117 (25.55)	58 (25.55)	59 (25.54)	0.998
Diabetes	43 (9.39)	20 (8.81)	23 (9.96)	0.674
Chronic obstructive pulmonary disease	0 (0.00)	0 (0.00)	0 (0.00)	-
Cause of artificial menopause	25 (5.46)	10 (4.41)	15 (6.49)	0.325
Other	222 (48.47)	116 (51.10)	106 (45.89)	0.264
History of fragility fracture	172 (37.55)	82 (36.12)	90 (38.96)	0.563
Spine	102 (22.27)	49 (21.59)	53 (22.94)	0.727
Hip	15 (3.28)	6 (2.64)	9 (3.90)	0.451
Proximal humerus	12 (2.62)	6 (2.64)	6 (2.60)	0.976
Distal radius	36 (7.86)	16 (7.05)	20 (8.66)	0.522
Distal ulna	3 (0.66)	1 (0.44)	2 (0.87)	0.575
Other	26 (5.68)	13 (5.73)	13 (5.63)	0.963
Compression fracture of thoracic spine, n (%)	382 (83.41)	190 (83.70)	192 (83.12)	0.900
Compression fracture of lumbar spine, *n* (%)	397 (86.68)	198 (87.22)	199 (86.15)	0.784
Lumbar spine BMD (g/cm^2^)	0.749 ± 0.101	0.745 ± 0.100	0.753 ± 0.104	0.419
T score	− 2.97 ± 0.83	− 3.00 ± 0.80	− 2.95 ± 0.85	0.529
Total hip BMD (g/cm^2^)	0.720 ± 0.096	0.726 ± 0.103	0.715 ± 0.089	0.216
T score	− 1.93 ± 0.78	− 1.87 ± 0.82	− 1.99 ± 0.74	0.084
Femoral neck BMD (g/cm^2^)	0.633 ± 0.098	0.635 ± 0.101	0.631 ± 0.095	0.670
T score	− 2.29 ± 0.75	− 2.26 ± 0.79	− 2.31 ± 0.70	0.501
β-CTX (ng/mL)	0.496 ± 0.244	0.493 ± 0.257	0.498 ± 0.231	0.830
P1NP (ng/mL)	57.90 ± 25.56	57.42 ± 26.35	58.36 ± 24.81	0.694
25(OH)D (ng/mL)	24.88 ± 9.78	25.12 ± 9.95	24.65 ± 9.62	0.609
Calcium (mmol/mL)	2.37 ± 0.11	2.37 ± 0.11	2.37 ± 0.11	0.823
Phosphate (mmol/mL)	1.24 ± 0.49	1.24 ± 0.15	1.25 ± 0.68	0.785
Creatinine clearance rate (mL/min)	79.64 ± 18.75	79.23 ± 19.27	80.04 ± 18.26	0.643

*ZOL* zoledronic acid, *β-CTX* C-terminal telopeptide of type 1collagen, *P1NP* procollagen 1 N-terminal peptide, *25(OH)D* 25-hydroxyvitamin D

### Changes of BMD and bone turnover biomarkers between two groups

**Fig. 2:**
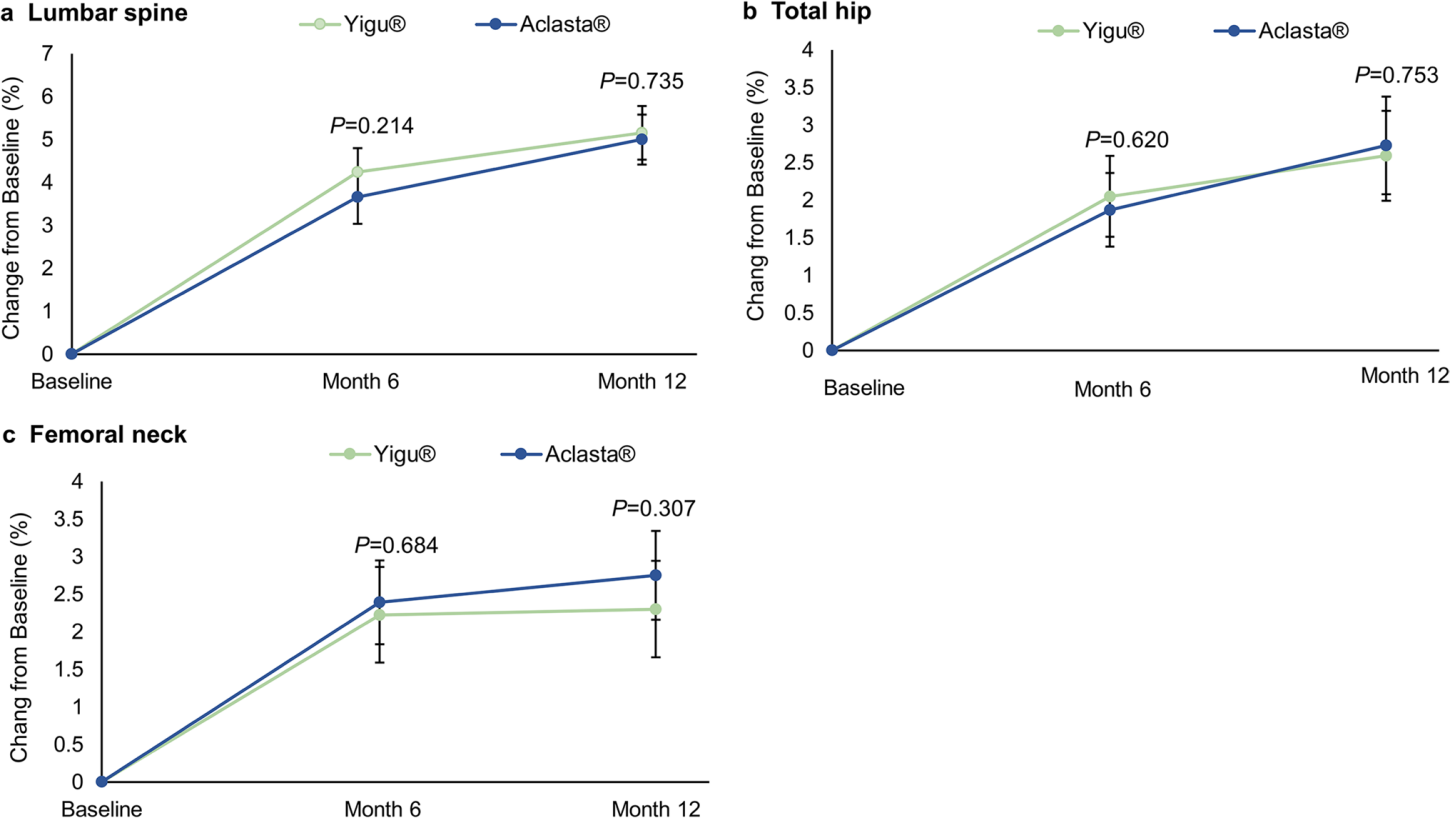
Mean changes in BMD over time. **a** Percentage change of BMD at lumbar spine in Yigu® and Aclasta® during the 12 months of treatment. **b** Percentage changes of BMD at total hip in Yigu® and Aclasta® during the 12 months of treatment. **c** Percentage changes of BMD at femoral neck in Yigu® and Aclasta® during the 12 months of treatment

On the basis of the FAS, at 6 months, the increases of BMD at lumbar spine, total hip, and femoral neck were 4.13% (95% CI: 3.57 to 4.68), 2.05% (95% CI: 1.51 to 2.59), and 2.22% (95% CI: 1.58 to 2.85) in Yigu® group and 3.60% (95% CI: 2.97 to 4.23), 1.87% (95% CI:1.38 to 2.36), and 2.39% (95% CI: 1.83 to 2.95) in Aclasta® group (all *P* < 0.001 vs baseline). There was no difference in BMD changes at 6 months between two groups (Fig. 2).

At 12 months, BMD at lumbar spine, total hip, and femoral neck increased by 5.15% (95% CI:4.52 to 5.77), 2.59% (95% CI: 1.99 to 3.19), and 2.30% (95% CI: 1.66 to 2.94) after Yigu® treatment and 5.00% (95% CI: 4.42 to 5.58), 2.73% (95% CI: 2.08 to 3.38), and 2.75% (95% CI: 2.16 to 3.34) after Aclasta® treatment (all *P* < 0.001 vs baseline). No difference in BMD changes was observed at lumbar spine (*P* = 0.735), total hip (*P* = 0.753), and femoral neck (*P* = 0.307) between these two groups (Fig. 2). At month 12, the mean difference of percentage change of BMD at lumbar spine from baseline between two groups was 0.15% (95% CI: − 0.71 to 1.00). The 95% CI of (− 0.71, 1.00) was well within the pre-specified equivalence boundaries (− 1.5, 1.5) (Table 2), indicating that Yigu® had the same efficacy as Aclasta® in the treatment of postmenopausal osteoporosis.

**Table 2:** Between-treatment comparison in percentage change in BMD at month 12

	Treatment	Mean change (%)	Mean % difference (95% CI)	*P* value
Lumbar spine	Yigu®	5.15	0.15 (− 0.71, 1.00)	0.735
	Aclasta®	5.00		
Total hip	Yigu®	2.59	− 0.14 (− 1.02, 0.74)	0.753
	Aclasta®	2.73		
Femoral neck	Yigu®	2.30	− 0.45 (− 1.32, 0.42)	0.307
	Aclasta®	2.75		

**Fig. 3:**
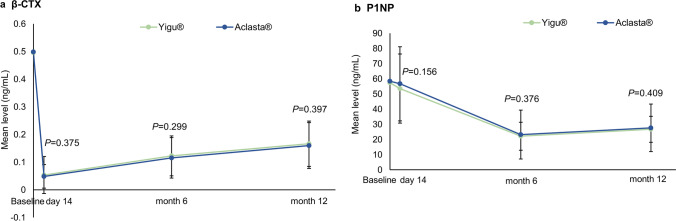
Mean changes in bone turnover biomarkers over time. **a** Changes of serum β-CTX levels in Yigu® and Aclasta® during the 12 months of treatment. **b** Changes of serum P1NP levels in Yigu® and Aclasta® during the 12 months of treatment. Results were shown as mean ± SD

Furthermore, no difference in percentage changes of β-CTX and P1NP was observed between two groups after 14 days and 6 or 12 months of treatment (Fig. 3).

### Changes of BMD and bone turnover biomarkers in the whole sample

As for the whole sample, the mean BMD significantly increased at lumbar spine (5.07%; 95% CI: 4.65 to 5.50), total hip (2.66%; 95% CI: 2.22 to 3.10), and femoral neck (2.52%; 95% CI: 2.09 to 2.96) (all *P* < 0.001 vs baseline) after 12 months of ZOL treatment. In addition, lumbar spine BMD rapidly and significantly improved after 6 months of ZOL treatment (*P* < 0.001 vs baseline).

The mean serum levels of β-CTX decreased rapidly and significantly by 87.86% at 14 days of ZOL treatment, while the decline in P1NP was relatively late which decreased significantly by 56.42% until 6 months of ZOL treatment. Both β-CTX and P1NP maintained below the value of baseline during the whole observation period.

### Safety

The incidence of all AEs was 88.55% and 90.04% in Yigu® and Aclasta® groups (*P* = 0.651) during the observational period, respectively (Table 3). Serious AEs (SAEs) were reported in 10 subjects (4.41%) in the Yigu® group and 13 subjects (5.63%) in the Aclasta® group (*P* = 0.670), including fracture, hypocalcemia, type 2 diabetes mellitus, and subarachnoid hemorrhage. One participant in Yigu® group was adjudicated as atrial fibrillation, and one subject in Aclasta® group had arrhythmia. No cases of osteonecrosis of the jaw and atypical fractures in both Yigu® and Aclasta® groups were reported or confirmed by adjudication. Except that the incidence of headache in Yigu® group was higher than that in Aclasta® group (16.74% vs 9.52%, *P* = 0.026), there was no statistical difference in the incidence of major AEs between the two groups, such as pyrexia, arthralgia, bone pain, myalgia, and upper respiratory tract infection. Similar to the overall AEs, the incidence of AEs during acute phase within 14 days of ZOL treatment was 85.46% and 84.42% in Yigu® and Aclasta® group, respectively (*P* = 0.795).

**Table 3:** Adverse events during the treatment

Events	Yigu® (*n* = 227) (%)	Aclasta® (*n* = 231) (%)	*P* value
Any adverse event	201 (88.55)	208 (90.04)	0.651
Any adverse event within 14 days of treatment	194 (85.46)	195 (84.42)	0.795
Any serious adverse event	10 (4.41)	13 (5.63)	0.670
Main adverse events			
Pyrexia^a^	146 (64.32)	158 (68.40)	0.374
Fatigue	47 (20.70)	47 (20.35)	1.000
Arthralgia	45 (19.82)	47 (20.35)	0.908
Myalgia	38 (16.74)	31 (13.42)	0.361
Dizziness	38 (16.74)	22 (9.52)	0.026
Back pain	33 (14.54)	37 (16.02)	0.698
Bone pain	29 (12.78)	40 (17.32)	0.193
Headache	28 (12.33)	31 (13.42)	0.781
Upper respiratory tract infection	18 (7.93)	18 (7.79)	1.000
Nausea	16 (7.05)	18 (7.79)	0.859
Anorexia	16 (7.05)	15 (6.49)	0.854
Diarrhea	13 (5.73)	9 (3.90)	0.390
Cough	10 (4.41)	14 (6.06)	0.531

^a^Axillary temperature: ≥ 37.3 °C

## Discussion

This multicenter and randomized controlled prospective study demonstrated that the infusion of 5 mg of Yigu® was equivalent to Aclasta® for the improvement of BMD at lumbar spine (primary endpoint). Meanwhile, similar efficacy results were observed for Yigu® and Aclasta® in increasing BMD at proximal femur and decreasing bone turnover biomarkers (secondary endpoints). Moreover, this study also revealed the similarity in safety of Yigu® compared with Aclasta®.

In this study, the BMD at lumbar spine, total hip, and femoral neck increased significantly from baseline in two ZOL groups, and the change of BMD in Yigu® group was comparable to that in Aclasta® group. Consistent with the results of our study, annual infusion of 5 mg ZOL was demonstrated to lead an increased BMD at lumbar spine and proximal hip in postmenopausal women with osteoporosis [7, 10–12]. In this study, serum β-CTX levels decreased rapidly after 14 days of ZOL treatment, followed by a decrease in serum levels of P1NP after 6 months of ZOL treatment. The effects of ZOL on bone turnover were also consistent with results of HORIZON-PFT and ZONE study [7, 10]. A systematic review concluded that 6 years of treatment of ZOL would reduce clinical vertebral fractures (HR, 0.41, CI: 0.22 to 0.75) and nonvertebral fractures (HR, 0.66, CI: 0.51 to 0.85) in women with osteopenia or osteoporosis [13]. A review reported a beneficial effect on survival of ZOL in addition to decreased fracture risk [14]; however, other large meta-analysis in 101,642 patients suggested that ZOL treatment was not associated with lower mortality risk [15]. Thus, the effects of ZOL on fracture incidence and survival rate in Chinese patients with osteoporosis were worthy of further study.

Bisphosphonates are the most widely used drugs for treatment of postmenopausal osteoporosis. ZOL, as one of the nitrogen-containing bisphosphonates (N-BPs), selectively targets bone resorption of osteoclasts by potently inhibiting FPP synthase [16]. Three kinds of N-BPs, including ZOL, alendronate, and risedronate, could not only increase BMD, but also decrease the incidence of osteoporotic bone fracture [17]. Previous study had proved a higher affinity of ZOL to hydroxyapatite than other BPs [18]. Meta-analysis confirmed that ZOL seemed to be the most effective BPs in reducing vertebral fracture, nonvertebral fracture, hip fracture, and any fracture in postmenopausal osteoporosis [19]. Zoledronate could reduce the risk of hip, vertebral, and nonvertebral fractures by 27–46%, as opposed to ibandronate, which decreased vertebral fracture risk, but was ineffective in decreasing hip or nonvertebral fractures risk [20]. Meanwhile, HORIZON study [21] had indicated that compared to risedronate, ZOL could more significantly improve BMD at lumbar spine (4.06% vs 2.71%), total hip (1.65% vs 0.45%), and femoral neck (1.45% vs 0.39%). Moreover, ZOL had a longer half-life time in bone, which could continuously reduce bone loss [22–25]. Treatment with annual infusion of ZOL was convenient and conducive to improve the compliance and persistence of osteoporosis patients and could still play a role for a long time after discontinuation [26]. Two randomized extensions of the HORIZON-PFT demonstrated that after 3 years of annual ZOL infusion, many patients would discontinue ZOL therapy up to 3 years and could still gain substantial residual benefit of ZOL [27, 28].

ZOL was generally well tolerated during 12 months of treatment; the overall safety profiles of Yigu® and Aclasta® groups were similar in this study. The most common AE was pyrexia in Yigu® and Aclasta® groups, but no difference in the incidence of AEs was observed between two groups. There was a lower frequency of SAEs in our study compared with prior study concerning ZOL treatment [27, 29, 30]; this discrepancy may attribute partly to the shorter follow-up period in our study (1 year versus ≥ 2 years), in which several AEs have not progressed to SAEs yet. The acute-phase reactions (APRs), characterized by transient mild-to-moderate influenza-like symptoms, were well resolved by antipyretic and antipyretic analgesic medications within 1 week of onset in our study. The occurrence of pyrexia in this study was consistent with that previously reported in Chinese postmenopausal women with osteoporosis receiving ZOL treatment [31–33], but higher than what reported in HORIZON-PFT [7], which was probably owing to the ethnic differences in ARPs to intravenous administration of ZOL [34]. Of note, no events of osteonecrosis of the jaw and atypical fracture were observed in this study, which was likely due to the short period of ZOL treatment in this study.

This was the first large-scale post-marketing study of ZOL in China. This study confirmed the clinical consistency in efficacy and safety of Yigu® and Aclasta® in postmenopausal women with osteoporosis with the equivalence analysis. In addition, this study also illustrated the efficacy and safety of both Yigu® and Aclasta® in increasing BMDs and decreasing bone turnover biomarkers in the whole sample. No previously unidentified AEs were reported during the 12-month observational period. However, this study had some limitations. First, we did not set up a placebo control group. Second, BMDs were measured by two distinct types of DXA in different hospitals, which might result in bias of results about BMD changes. Additionally, the treatment and observational period of this study were relatively short, which was difficult to observe the effects of treatment on incidence of fracture. Therefore, larger and longer clinical studies were needed to evaluate the effects of ZOL on incidence of bone fracture in Chinese population.

In conclusion, zoledronic acid can significantly inhibit bone loss and increase BMD of Chinese postmenopausal women with osteoporosis. The efficacy and safety of Yigu® are similar to those of Aclasta® in postmenopausal women with osteoporosis.
